# Three-Dimensional PNP–FEM of a Layered IPMC Artificial Skin Under Finger-like Sliding for Robotic Tactile Interfaces

**DOI:** 10.3390/s26102930

**Published:** 2026-05-07

**Authors:** Montassar Aidi Sharif

**Affiliations:** Artificial Intelligence Engineering Technology Department, Technical Engineering College for Computer and AI/Kirkuk, Northern Technical University (NTU), Mosul 41002, Iraq; msharif@ntu.edu.iq; Tel.: +964-773-057-5786

**Keywords:** artificial skin (AS), IPMC sensor, shear sensing, Layered IPMC, Poisson–Nernst–Planck (PNP), robotic tactile interfaces, finite element analysis (FEA), COMSOL, soft robotics

## Abstract

Robotic tactile interfaces involving artificial skins often experience sliding contact conditions. At sliding interfaces, frictional loading, tangential stress, and impending slip dominate sensing behavior. This work demonstrates three-dimensional finite element (3D-FE) and Poisson–Nernst–Planck (PNP) modeling of layered ionic polymer–metal composite (IPMC) artificial skin under finger-like reciprocating sliding contact. The layered structure consists of a Nafion-based IPMC core sandwiched between thin upper and lower electrodes. A rigid acrylic slider is used to simulate reciprocating finger motion relative to the surface of the IPMC skin. A time-dependent contact mechanics model is first utilized to simulate temporal variations in normal and tangential contact fields for various coefficients of friction. Electrochemical response is then determined in COMSOL Multiphysics by coupling ion transport and electrostatics in a PNP framework to predict the output sliding current. Parametric studies are used to investigate the dependence of sensor response on the coefficient of friction, reciprocating history, layer geometry, and transport parameters. From the results, it can be noted that the resulting parameter offers a robust and physically meaningful description of the magnitude of contact-induced shear stress under multi-mode loadings, yet retaining the capability of responding to the presence of friction-induced mechanical excitation. The current model is aimed at dynamic shear sensitivity detection in sliding contacts. It is not designed for texture discrimination or fragment identification tasks. Thus, the current study demonstrates an important coupling parameter for 3D IPMC sensor models under contact and sets up a framework for enhanced electro-chemo-mechanical modeling of soft ionic tactile sensors.

## 1. Introduction

Robotic tactile interfaces and synthetic skins are increasingly essential for functioning in contact-intensive environments where sliding, tangential loading, and initial slip significantly affect sensing performance [1,2,3]. In robotic fingers, compliant grippers, and soft contact pads, tactile interaction transcends conventional indentation or bending; it frequently encompasses friction-induced contact, transient slip, and distributed interfacial shear [4,5,6]. An effective tactile material for robotic applications must be compliant, lightweight, and able to transform mechanically induced surface interactions into quantifiable electrical outputs under dynamic contact conditions [7].

Ionic polymer–metal composites (IPMCs) have garnered significant interest as a material platform for soft sensing due to their integration of mechanical flexibility and electromechanical transduction within a low-voltage soft structure [8,9,10]. An IPMC usually has an ionomer core, usually Nafion, between two conductive electrodes. This lets ions move around inside the hydrated polymer and turn into an electrical response [11,12,13]. Because of these traits, IPMCs have been looked into for use in soft robotics, biomimetics, flexible sensing, and smart systems that can do more than one thing [14,15,16,17].

Depending on the readout scheme and excitation mode, IPMC outputs in sensing mode are often reported as open-circuit voltage, short-circuit current, impedance variation, or self-sensing electrical response [18,19,20]. Transient current is especially useful for dynamic tactile events because it is directly related to the rate at which the charge on the electrodes changes over time. This means that it can show the start, development, and relaxation of mechanically induced ionic redistribution [18,20]. This makes current-based readout very useful for sliding tactile interaction, where sudden changes in contact state and tangential traction are the most important things to look at.

Even though a lot of work has been done on IPMC actuation and sensing, most of the previous research on sensing has focused on strip or cantilever geometries that bend [21,22]. These studies have significantly elucidated electromechanical coupling, ionic migration, and transient response mechanisms [23,24,25,26]; however, they do not comprehensively represent the frictional and shear-dominated conditions experienced in robotic tactile interaction. In real-world robotic manipulation, finger-like structures often move back and forth across a surface that they touch, creating normal pressure, tangential traction, and sliding-induced interfacial effects that are more like tactile exploration and slip-related sensing than just bending alone [27,28,29]. This observation leads to the creation of IPMC modeling frameworks that clearly deal with sliding contact and friction that goes back and forth.

From a physics perspective, IPMC behavior is typically explained by considering the hydrated ionomer as a charged medium with mobile counter-cations and fixed anionic groups [11,24]. In this framework, the electric field is controlled by electrostatics and ionic transport is described by migration–diffusion relations. This results in coupled Poisson–Nernst–Planck (PNP) formulations that can capture transient charge accumulation and relaxation across the polymer thickness [23,25,30,31]. Because these physics-based models connect material properties, geometric configuration, and transport parameters to quantifiable electrical outputs, they are especially appealing for simulation-driven design. While more sophisticated formulations might include poromechanical, steric, or multiphase effects [31,32], standard PNP modeling continues to be a physically significant and computationally feasible foundation for transient electrochemical sensing analysis.

Simultaneously, realistic tactile interaction under sliding and frictional loading can be practically represented using finite element contact mechanics [33]. In addition to internal ionic transport, the evolution of contact pressure, tangential traction, friction level, and sliding history during surface interaction all influence the sensing response for layered artificial skin concepts. Therefore, a modeling approach that integrates electrochemical transport and time-dependent contact mechanics is ideal for examining how a soft ionic layer functions as an artificial skin under robotic touch.

Additionally, this approach logically expands upon our earlier work. The current author and colleagues have previously studied omnidirectional tubular IPMC sensing [34], torsional IPMC sensing [35], bioinspired pressure-gradient sensing [36,37], and soft smart sensing for robotics applications [38]. These studies have created a more comprehensive foundation in soft sensor-oriented design and physics-based modeling. In contrast to those previous ideas, the current work focuses on a planar layered skin-like architecture under reciprocating sliding contact that resembles fingers. This is more in line with robotic tactile interfaces.

Motivated by the above facts, this paper develops a novel methodology utilizing 3D finite element analysis and PNP equations for the purpose of modeling an IPMC skin layer experiencing reciprocating sliding action resembling a finger. The model includes a Nafion ionomer sandwiched between two thin layers of upper and lower electrodes. An acrylic slider is considered in order to represent the reciprocating action of a robot finger on the skin surface. First, the problem of mechanics is solved based on time-dependent contact mechanics, considering friction to calculate the change in the normal and shear interfacial fields at various friction coefficients. Second, the electrochemical problem is solved in the context of Nafion ionomer through a PNP modeling approach to calculate the generated current in the sliding motion.

In the current paper, the suggested sensing function is mainly formulated as the dynamic shear-sensitive tactile sensor operating in the sliding contact regime, possibly applicable in the context of indicating imminent slip onset. Specifically, the model is developed for describing the process of transformation of the time-dependent tangential interaction and sliding status into electrical transients. At the current stage, the current approach is not designed to deal with problems such as texture recognition, piecewise estimation, and material property identification. The principal contributions of this study are as follows: (i) a finite element contact mechanics model for a multi-layered IPMC skin subject to cyclic touch–sliding–lift loading; (ii) a PNP-based electrochemical model induced by the externally applied shear stress field; and (iii) a friction-related parametric study elucidating the role of tangential conditions on transients in the tactile response.

## 2. Mathematical Modeling

The following information gives a mathematical formulation to evaluate current flow through a layered ionic polymer–metal composite (IPMC) artificial skin under a repeated finger touch–slide–lift motion. The model employed has been developed through two sequential time-dependent analyses: The first analysis involved the solution of contact mechanics to find the shear and frictional deformation levels in each layered structure during the contact and sliding cycle. The second analysis provided the electrochemical response characterizing the Nafion core using a Poisson–Nernst–Planck (PNP) model of coupling. This overall formulation of our IPMC design was based on previously reported IPMC electromechanical and electrochemical modeling in the literature [11,23,24,25,35].

### 2.1. Geometry, Domains, and Primary Fields

The proposed artificial skin (AS) consists of a rectangular layer of IPMC structure formed by three united domains: a bottom electrode $\Omega_{b}$ made of platinum, a Nafion-based ionomer core $\Omega_{n}$, and a top platinum electrode $\Omega_{t}$. These three united layers define the deformable IPMC sensor body

(1) $$\Omega_{m}=\Omega_{b}\cup \Omega_{n}\cup \Omega_{t}.$$

A rigid acrylic slider occupying domain $\Omega_{s}$ is positioned above the top electrode and is used to emulate the cyclic motion of a robotic finger over the skin surface. Frictional contact is established only when the slider is in its lowered state and in contact with the upper face of the top electrode.

The primary unknown fields are as follows:

- The mechanical displacement field u(x,t) and the corresponding stress field σ(x,t) in Study 1;
- The cation concentration c(x,t) and electric potential ϕ(x,t) in Study 2.

All analyses are carried out in the time domain.

### 2.2. Step 1: Time-Dependent Contact Mechanics

The IPMC sensor and the slider are modeled as nearly incompressible elastic solids, where the slider has prescribed translational motion. Under the quasi-static assumption adopted in the present work, the mechanical equilibrium equation in the deformable domains is written as follows:

(2) $$\nabla \cdot \sigma +b=0\;\mathrm{in}\;\Omega_{m},$$

where b denotes the body-force density. The infinitesimal strain tensor is defined as follows:

(3) $$\varepsilon =\frac{1}{2}\left(\nabla u+{\left(\nabla u\right)}^{T}\right),$$

and the constitutive relation is taken in linear elastic form,

(4) σ=C:ε,

where C is the elasticity tensor [24,25,33].

The mechanical model chosen for the current study is based on the small-strain continuum mechanics approach, which is commonly utilized in contact and deformation studies. For instance, the quasi-static equilibrium condition, infinitesimal strain measure, and linear elasticity assumption given in Equations (2)–(4) are not considered to be novel assumptions for this work, but rather are borrowed from the conventional theories of continuum mechanics and finite element-based contact modeling [33]. The unique feature of the current study is that rather than proposing novel kinematics and mechanical models, they are embedded into a layered IPMC touch–slide–lift sensing system.

The interfaces among the bottom electrode, Nafion core, and top electrode are assumed to be perfectly bonded. The lower surface of the bottom electrode is fixed to represent the attachment of the artificial skin (AS) to a rigid support. The slider is constrained against rotation and undergoes only prescribed translational motion.

Cyclic Touch–Slide–Lift Motion:

To simulate a dynamic finger-like tactile scanning process, we define the slider motion as a periodic multi-stage cycle:

1. Vertical downward motion—until it comes in contact;
2. Horizontal sliding on the artificial skin;
3. Vertical upward movement to break contact;
4. Horizontal motion during return while not adhered to the surface.

$g_{0}$ denotes the initial gap between the slider and the top electrode, $\delta_{p}$ is a small preload displacement introduced to ensure stable contact, and *s* is the horizontal sliding stroke. Let $t_{D}$, $t_{S}$, $t_{L}$, and $t_{R}$ denote the durations of touchdown, sliding, lifting, and return, respectively. The total cycle duration is

(5) $$T_{\mathrm{cyc}}=t_{D}+t_{S}+t_{L}+t_{R}.$$

To describe periodic repetition, a local cycle time is introduced as follows:

(6) $$\tau =t-\left\lfloor \frac{t}{T_{\mathrm{cyc}}}\right\rfloor T_{\mathrm{cyc}},\;0\leq \tau <T_{\mathrm{cyc}}.$$

The prescribed vertical displacement of the slider is then maintained:

(7) $$u_{z}^{\left(s\right)}\left(t\right)=\left\{\begin{array}{cc} -\left(g_{0}+\delta_{p}\right)\frac{\tau }{t_{D}},\; & 0\leq \tau <t_{D},\\ -\left(g_{0}+\delta_{p}\right),\; & t_{D}\leq \tau <t_{D}+t_{S},\\ -\left(g_{0}+\delta_{p}\right)\left(1-\frac{\tau -(t_{D}+t_{S})}{t_{L}}\right),\; & t_{D}+t_{S}\leq \tau <t_{D}+t_{S}+t_{L},\\ 0,\; & t_{D}+t_{S}+t_{L}\leq \tau <T_{\mathrm{cyc}}, \end{array}\right.$$

which represents the approach, maintained contact during sliding, lift-off, and detached return.

The prescribed horizontal displacement is defined as follows:

(8) $$u_{x}^{\left(s\right)}\left(t\right)=\left\{\begin{array}{cc} 0,\; & 0\leq \tau <t_{D},\\ s\;\frac{\tau -t_{D}}{t_{S}},\; & t_{D}\leq \tau <t_{D}+t_{S},\\ s,\; & t_{D}+t_{S}\leq \tau <t_{D}+t_{S}+t_{L},\\ s\left(1-\frac{\tau -(t_{D}+t_{S}+t_{L})}{t_{R}}\right),\; & t_{D}+t_{S}+t_{L}\leq \tau <T_{\mathrm{cyc}}. \end{array}\right.$$

The slider moves in contact with the artificial skin only during the sliding stage, whereas the return takes place in the detached state. This motion is intended to provide a transient excitation phase followed by a partial relaxation phase within each of the cycles.

Contact and Friction:

Frictional contact is imposed on the interface $\Gamma_{c}$ between the lower surface of the acrylic slider and the upper surface of the top electrode. The tangential traction $t_{T}$ satisfies the Coulomb friction law,

(9) $$∥t_{T}∥\leq \mu_{f}\;p_{n},$$

where $p_{n}$ is the normal contact pressure and $\mu_{f}$ is the friction coefficient [33]. Because contact is active only during the touchdown and sliding phases, the resulting normal and tangential tractions are strongly time-dependent and are expected to generate a more dynamic shear stimulus than in sustained-contact reciprocating motion.

The current work uses Coulomb friction to model the interface at first order, in order to focus on the impact of the level of friction on the transfer of tangential stresses and subsequent shear transduction. This selection offers a simple and easy-to-understand contact relationship in the context of the parameter sweep presented in this paper. It should be noted, nevertheless, that higher-order interface relationships, including regularized, rate-dependent, and adhesion-augmented friction, can describe other aspects of the slider–sensor interaction in greater detail.

Equivalent Shear Strain:

To characterize the intensity of contact-induced shear, an invariant equivalent shear strain is extracted from the mechanical solution as follows:

(10) $$\gamma_{\mathrm{eq}}\left(x,t\right)=\sqrt{2\left(\varepsilon_{xy}^{2}+\varepsilon_{yz}^{2}+\varepsilon_{xz}^{2}\right)}.$$

The shear-strain field corresponding to Equation (10) has been employed here as a scalar indicator for the magnitude of the contact-driven tensorial shear state. This quantity is formed using the non-diagonal elements of the infinitesimal strain tensor and serves as the mechanically derived variable for the electrochemical study that follows. This approach is in keeping with the scalar formulation of shear quantities based on tensorial strain variables in theories of multicomponent strain states [39,40,41]. In the current study, this variable has been selected for its ability to yield a concise, non-negative measure of shear for one-way transfer between Study 1 and Study 2.

### 2.3. Step 2: Electrochemical Model Based on the PNP Framework

The physical electrochemical model utilized in the current study is derived from existing physics-based electroactive polymer models that relate ion transport and electrostatics using a Poisson–Nernst–Planck framework. Consequently, the ion continuity equation, Nernst–Planck flux, Gauss’s law, and current definition approach presented in this work conform to the modeling strategy proposed in previous works on IPMC electromechanics and electrodynamics [11,23,24,25,35]. It is thus clear that the unique feature of the current study lies not in the derivation of a novel PNP model, but rather in its application to an artificial skin model subject to repeated touch–slide–lift mechanisms.

Ion Transport:

The conservation equation for the mobile counter-cation concentration is written as follows:

(11) $$\frac{\partial c}{\partial t}+\nabla \cdot N=0\;\mathrm{in}\;\Omega_{n},$$

where N is the ionic flux. The latter is defined using the Nernst–Planck relation

(12) $$N=-D\nabla c-z\;u_{m}\;F\;c\;\nabla \phi ,$$

where *D* is the diffusion coefficient, $u_{m}$ is the ionic mobility, *z* is the ionic valence, and *F* is Faraday’s constant [11,23,35].

Electrostatics:

The electric potential field is governed by Gauss’ law,

(13) $$-\nabla \cdot \left(\epsilon \nabla \phi \right)=\rho \;\mathrm{in}\;\Omega_{n},$$

where ϵ is the effective permittivity and ρ is the free charge density [23,24,25].

Mechanically Informed Effective Fixed-Charge Concentration:

To embed the mechanical response within a sequential one-way coupling with the electrochemical problem, this work introduces an effective fixed-charge concentration expressed as a function of the equivalent shear-strain field:

(14) $$c_{f}^{\mathrm{eff}}\left(x,t\right)=c_{f}\left(1-\alpha \;\gamma_{eq}\left(x,t\right)\right),$$

where $c_{f}$ is the baseline fixed-charge concentration and α is a coupling coefficient.

The relation in Equation (14) is introduced here as a phenomenological first-order coupling law rather than as a fully derived microscopic constitutive law. Its purpose is to provide a compact representation of the idea that mechanically induced shear may perturb the local electrochemical state of the hydrated ionomer and thereby modify the effective space-charge distribution entering the PNP model. The chosen form is the simplest nontrivial linearized ansatz around the reference state, since it (i) preserves the baseline fixed-charge level when $\gamma_{eq}=0$, (ii) introduces a monotonic shear-dependent perturbation under sliding contact, and (iii) remains suitable for stable one-way numerical coupling between the mechanical and electrochemical subproblems.

The parameter α should therefore be interpreted as a bounded sensitivity coefficient rather than a directly measured material constant in the present work. Its value was selected so that the perturbation of $c_{f}^{\mathrm{eff}}$ would remain within a physically meaningful small-signal regime over the full simulated range of $\gamma_{eq}$ and would not lead to nonphysical negative values of the effective fixed-charge concentration. In this sense, the adopted law is intended for exploratory model-level transduction analysis, while its precise calibration should be addressed in future experimental work.

Using Equation (14), the space-charge density is defined as follows:

(15) $$\rho =F\left(zc-c_{f}^{\mathrm{eff}}\right).$$

Accordingly, Equations (11)–(15) define the coupled PNP system solved in Study 2.

### 2.4. Boundary and Initial Conditions

Let $\Gamma_{n,b}$ and $\Gamma_{n,t}$ denote the lower and upper Nafion interfaces adjacent to the bottom and top electrodes, respectively.

Blocking Electrode Condition:

The metallic electrodes are assumed to be blocking to ionic transport, which implies

(16) $$n\cdot N=0\;\mathrm{on}\;\Gamma_{n,b}\cup \Gamma_{n,t}.$$

For simplicity, the remaining external boundaries of the Nafion core are also taken as zero-flux boundaries unless otherwise stated.

Short-Circuit Electrical Condition:

A short-circuit configuration is imposed by setting the same electric potential on both Nafion/electrode interfaces:

(17) $$\phi =0\;\mathrm{on}\;\Gamma_{n,b},\;\phi =0\;\mathrm{on}\;\Gamma_{n,t}.$$

Initial Conditions:

The electrochemical simulation starts from a uniform concentration and zero electric potential:

(18) $$c\left(x,0\right)=c_{0},\;\phi \left(x,0\right)=0.$$

### 2.5. Current Readout

The transient current is obtained from the electrode boundaries using the displacement-current formulation. The electric displacement vector is

(19) D=−ϵ∇ϕ,

and the displacement current density is

(20) $$J_{d}=\frac{\partial D}{\partial t}.$$

The current measured on a selected electrode boundary $\Gamma_{e}$ is then

(21) $$I\left(t\right)=\int_{\Gamma_{e}}J_{d}\cdot n\;dA.$$

Equivalently, if the surface charge density is defined as

(22) $$\sigma_{s}\left(x,t\right)=-n\cdot D{|}_{\Gamma_{e}},$$

then the total electrode charge is

(23) $$Q_{e}\left(t\right)=\int_{\Gamma_{e}}\sigma_{s}\;dA,$$

and the current satisfies

(24) $$I\left(t\right)=\frac{dQ_{e}}{dt}.$$

As can be seen from Equations (18)–(24), the current readout model is similar to the one used in the earlier investigations of IPMC sensors, where the transient electrical response was analyzed based on the time dependence of charge and current at the electrodes’ boundaries [18,20,35]. This model is maintained in the current study, since the sensed parameter is the transient short-circuit current.

### 2.6. Coupling Strategy

Sequential one-way coupling is used to carry out the electromechanical transduction. The equivalent shear-strain field $\gamma_{\mathrm{eq}}\left(x,t\right)$ is calculated from the mechanical solution in Study 1 after the layered IPMC artificial skin is subjected to cyclic touch–slide–lift motion. The effect of sliding-induced shear is introduced into the electrochemical problem in Study 2 by using this imported field to define the effective fixed-charge concentration through Equation (14). After solving the PNP system in the Nafion core, Equation (21) yields the transient sensor output as the current on the electrode boundary. Adopting a cyclic contact–release sequence is meant to better capture the dynamic nature of IPMC sensing, where robotic tactile scanning repeatedly involves partial relaxation and transient excitation.

## 3. Finite Element Implementation in COMSOL

The proposed framework was implemented in COMSOL Multiphysics v6.1 using two sequential time-dependent studies. Study 1 was used to solve the mechanical problem of the layered IPMC artificial skin under cyclic finger-like touch–slide–lift motion. Study 2 was then used to solve the electrochemical response in the Nafion core by coupling the *Transport of Diluted Species* and *Electrostatics* interfaces within a Poisson–Nernst–Planck (PNP) framework. The equivalent shear-strain field obtained from Study 1 was imported into the electrochemical analysis, and the transient current response was extracted from the electrode boundaries by integrating the normal displacement current density.

### 3.1. Geometry Construction and Domain Arrangement

**Figure 1:**
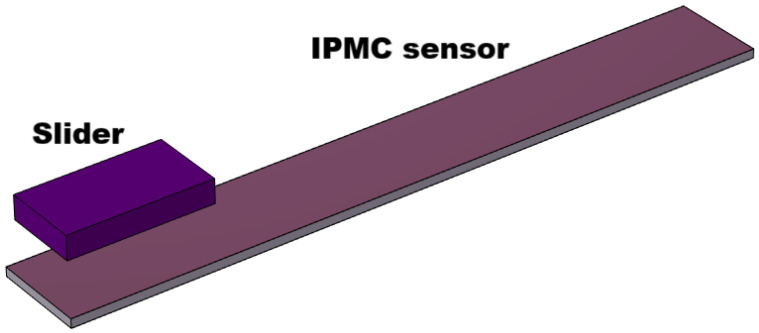
3D schematic of the proposed modeled system, showing the slider and the IPMC artificial skin.

The three-dimensional model consists of a layered IPMC artificial skin and a rigid acrylic slider. The IPMC body is formed by stacking three bonded rectangular layers in the thickness direction, namely, the bottom electrode, the Nafion core, and the top electrode. The acrylic slider is positioned above the top electrode with an initial gap and remains a separate body in order to define a contact pair with the upper IPMC surface. A three-dimensional view of the overall model is shown in Figure 1, where the slider and the IPMC sensor are illustrated before the cyclic touch–slide–lift interaction is applied.

In the baseline configuration, the layered IPMC has a length of 15 mm and a width of 2 mm. The bottom and top electrodes each have a thickness of 0.005 mm, whereas the Nafion core has a thickness of 0.190 mm, resulting in a total IPMC thickness of 0.200 mm. The acrylic slider has dimensions 3×1.6×0.5 mm^3^ and is initially placed 1 mm above the top electrode.

**Figure 2:**
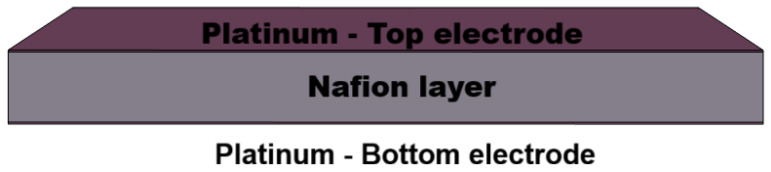
Schematic representation of the IPMC sensor.

A schematic view of the internal layered structure of the IPMC sensor is shown in Figure 2. This representation highlights the physical composition adopted in the finite element model, where the two platinum electrodes bound the Nafion ionomer layer and form the electromechanically active sensing structure.

### 3.2. Study 1: Solid Mechanics with Cyclic Frictional Contact

Study 1 was solved using the *Solid Mechanics* interface. The three-layer IPMC body was treated as deformable, whereas the acrylic slider was modeled using the *Rigid Material* feature with prescribed translational motion and constrained rotations. The lower surface of the bottom electrode was fixed to represent attachment of the artificial skin to a rigid support. Perfect bonding was assumed between the two electrodes and the Nafion core.

Contact between the lower surface of the acrylic slider and the upper surface of the top electrode was implemented using a pair-based *Contact* feature. Coulomb friction was enabled with a friction coefficient $\mu_{f}$ in order to generate tangential traction and sliding-induced shear within the layered IPMC body. The successful parametric sweep reported in this work covered $\mu_{f}=0.001$–0.30.

The reason for selecting this range of friction was to explore a wide range from the case of very low tangential coupling to one of moderate tangential coupling. This was meant not to simulate a particular case of surface interaction but, rather, to explore the mechanical and electrical behavior of the system when the slider–sensor interface changes from a regime of lubrication to one where friction dominates.

To better reflect the dynamic nature of IPMC sensing, the slider was driven by a cyclic four-stage motion. In each cycle, the slider first moved vertically downward until contact was established, then slid horizontally across the top surface while maintaining contact, then moved upward to release contact, and finally returned horizontally to the starting position while detached from the surface. In COMSOL, this motion was implemented using time-dependent piecewise displacement functions with a local cycle time

$$\tau =\mathrm{mod}(t,T_{\mathrm{cyc}}),$$

where $T_{\mathrm{cyc}}$ is the total cycle duration.

**Figure 3:**
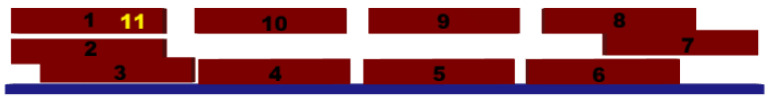
Schematic sequence of the cyclic touch–slide–lift motion of the slider. The numbered positions represent the main stages of touchdown, sliding in contact, lift-off, and detached return to the starting position.

The full sequence of the adopted slider motion is illustrated in Figure 3. The figure shows the discrete stages of touchdown, contact sliding, lift-off, and return, and therefore provides a direct visual interpretation of the imposed tactile-scanning cycle used throughout the simulations.

The vertical and horizontal slider motions were prescribed through the piecewise functions corresponding to Equations (7) and (8). In the baseline configuration, the cycle durations were selected as $t_{D}=0.15$ s for touchdown, $t_{S}=0.35$ s for sliding in contact, $t_{L}=0.10$ s for lift-off, and $t_{R}=0.25$ s for detached return, giving a total cycle duration of $T_{\mathrm{cyc}}=0.85$ s. Unless otherwise stated, three cycles were simulated, leading to a total analysis time of $t_{\mathrm{end}}=2.55$ s.

The above-mentioned geometric and kinematic parameters are meant to model the real-world case of robotic tactile scanning of the material sample. In particular, the sizes of the slider and the sliding distance were chosen in such a way that the area of contact always stayed within the active surface of the sensor during the phase of touching and sliding. The choice of the touching–slide–lifting cycle was aimed at imitating the process of cyclic tactile probing with relaxation at intermediate steps. As a result, the set time for each phase makes it possible to clearly define the moments of touchdown, touch slide, detachment, and lifting back. The small preload was used to make sure that the contact was established reliably but caused only slight indentation of the multi-layered material structure.

The equivalent shear strain,

(25) $$\gamma_{\mathrm{eq}}\left(x,t\right)=\sqrt{2\left(\varepsilon_{xy}^{2}+\varepsilon_{yz}^{2}+\varepsilon_{xz}^{2}\right)},$$

was computed from the mechanical solution and exported for use in Study 2.

### 3.3. Study 2: PNP Electrochemistry in the Nafion Core

Study 2 was solved only in the Nafion domain by coupling the *Transport of Diluted Species* and *Electrostatics* interfaces. Although the electrode layers were retained in the geometry for the mechanical problem, electrochemically they were represented through boundary conditions imposed on the upper and lower Nafion interfaces.

In the transport interface, the mobile cation concentration was solved using the Nernst–Planck flux including diffusion and electromigration. In the electrostatics interface, the electric potential was obtained from Gauss’s law, with the space-charge density defined by the evolving cation concentration and the mechanically informed effective fixed-charge concentration. The imported equivalent shear-strain field $\gamma_{\mathrm{eq}}\left(x,t\right)$ from Study 1 was used to define

(26) $$c_{f}^{\mathrm{eff}}\left(x,t\right)=c_{f}\left(1-\alpha \;\gamma_{\mathrm{eq}}\left(x,t\right)\right),$$

The coupling relationship shown in Equation (26) is utilized here as a phenomenological mapping of the first order between the equivalent shear strain imported through mechanics and the fixed-charge state. Here, in the current study, the primary role of this equation is not to serve as a calibrated constitutive law but as a perturbation on the reference value of fixed charges under shearing action induced by sliding. The parameter α is thus considered as a sensitivity factor that ensures physically sensible values of $c_{f}^{\mathrm{eff}}$ for any value of $\gamma_{eq}$. The equation entered the space-charge expression

(27) $$\rho \left(x,t\right)=F\left(zc\left(x,t\right)-c_{f}^{\mathrm{eff}}\left(x,t\right)\right).$$

Blocking-electrode conditions were imposed by specifying zero normal ionic flux at the Nafion/electrode interfaces. A short-circuit electrical condition was imposed by assigning the same electric potential to the upper and lower Nafion interfaces. The electrochemical problem was solved over the same time interval as Study 1.

### 3.4. Meshing, Time Stepping, and Solver Settings

The mesh was refined in the thickness direction of the layered IPMC. Additional refinement was also applied in the contact region and near the upper and lower Nafion interfaces, where stronger gradients in concentration and electric potential were expected. The time stepping was selected to capture the touchdown, sliding, lift-off, and relaxation phases within each cycle. In the reported simulations, a baseline time discretization of

$$\mathrm{range}(0,0.001,t_{\mathrm{end}})$$

was employed for the determination of the touchdown, in-contact sliding, lift-off, and detached return stages of each cycle. Study 1 was solved first for each value of the friction coefficient; then, Study 2 was solved for the corresponding equivalent shear-strain field obtained from Study 1.

The adopted mesh size and time step were chosen to ensure a stable numerical solution, along with adequate spatial resolution for the most important regions concerning the contact transfer and electrochemical processes. These specifications are provided in the revised manuscript.

### 3.5. Current Extraction

The transient current was extracted from either the upper or lower Nafion/electrode interface by integrating the normal component of the displacement current density:

(28) $$I\left(t\right)=\int_{\Gamma_{e}}J_{d}\cdot n\;dA,\;J_{d}=\frac{\partial D}{\partial t},\;D=-\epsilon \nabla \phi .$$

This is equivalent to computing the time derivative of the total electrode charge. In COMSOL postprocessing, Equation (28) was evaluated by surface integration of the normal displacement current density on the selected electrode boundary. The opposite electrode yielded approximately the same magnitude with the opposite sign.

### 3.6. Baseline Model Parameters

The key geometric, loading, and material inputs considered in the mathematical formulation and COMSOL simulation are listed in Table 1. In the current research work, the ambient conditions were deliberately kept constant so that the influence of only contact mechanics, friction-dependent shear coupling, and electrochemical transduction could be considered, without any further ambiguity arising due to changing ambient factors. For instance, the calculations were done by keeping the ambient temperature at T=290 K. It should be taken into account that this temperature is not meant to imply the independence of the response from changes in temperature but represents merely a reference state for the calculations. The inputs in Table 1 can be regarded as default parameters associated with an optimal robotic tactile scanning operation. For instance, the slider size, displacement range, cycle time, and preloading were selected to facilitate consistent transient contact, prolonged on-skin sliding, and eventual separation inside the effective sensing area. The immovable lower boundary symbolizes the artificial skin’s secure mounting onto a hard surface, whereas the open-circuit assumption corresponds to the adopted sensing modality in the current work.

## 4. Results and Discussion

In this section, the predicted mechanical and electrical responses of the layered IPMC artificial skin under cyclic touch–slide–lift motion are analyzed. The results were obtained using the two-stage workflow described in Section 2 and Section 3. In Study 1, the frictional contact problem was solved for

$$\mu_{f}=\left\{0.001,0.005,0.007,0.01,0.05,0.07,0.1,0.2,0.3\right\},$$

using the prescribed cyclic slider motion given by Equations (7) and (8). In Study 2, the equivalent shear-strain field imported from the mechanical solution was used to solve the coupled PNP problem in the Nafion core, and the electrical output was finally expressed in terms of transient current extracted from the electrode boundary through Equation (28).

The results are interpreted in the framework of dynamic shear-sensitive tactile sensing under sliding contact. In this interpretation, the layered IPMC structure acts primarily as a transient tactile transducer, meaning that the strongest electrical response is expected during time-varying contact events, tangential loading, and sliding-state transitions rather than under quasi-static loading alone. The present results are therefore discussed with particular emphasis on friction-dependent shear transfer, transient current generation, and the potential relevance of the predicted response to incipient slip indication.

The results are presented in the framework of the dynamic sensing hypothesis of the present work, which implies that the layered IPMC artificial skin acts primarily as a transient or dynamic tactile transducer, i.e., the strongest electrical response is expected in the case of time-dependent contact events rather than quasi-static loading conditions. Along these lines, the presentation of the results is divided into four parts: First, the cyclic motion used in the experiments is described as the common basis for the presentation of the results that follow. Second, the dependence of the response on friction is addressed in the framework of the equivalent shear-strain field. Third, the predicted response in the form of the generated current is addressed for the full range of friction values used in the experiments. Fourth, the mechanical–electrical correlations are addressed in a compact form in order to facilitate the interpretation of the results in the framework of the transduction mechanism.

### 4.1. Input Motion and Cycle Definition

**Figure 4:**
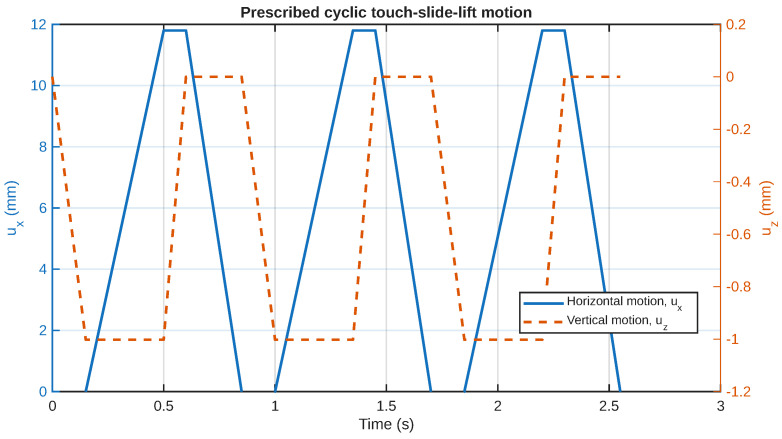
The prescribed cyclic touch–slide–lift motion of the slider: $u_{x}\left(t\right)$ is the horizontal displacement and $u_{z}\left(t\right)$ is the vertical displacement. The motion itself is touchdown, then sliding in contact after that lift-off, and finally the detached return.

The imposed motion of the slider establishes a time structure for the entire process of sensing and, therefore, establishes a reference frame for understanding the observed mechanical and electrical responses. As shown in Figure 4, the input motion is not a simple sinusoid; rather, it is a cyclic process consisting of a series of touches, slides, and lifts. In each cycle of this process, the acrylic slider moves downward until it touches the top electrode, then slides horizontally while maintaining contact, then lifts off vertically, and finally slides horizontally again, but this time while not in contact. Note that this input motion was selected to more closely mimic a dynamic process of tactile scanning and to allow a partial electrochemical relaxation between touches.

The piecewise nature of the proposed motion indicates that the sensor is primarily being excited during the touchdown and sliding phases. On the other hand, the lift-off and detached return phases are responsible for reducing or eliminating contact-induced shear. This is an important characteristic because it is compatible with the IPMC sensor’s dynamics. This is because transient excitation is expected to produce stronger currents than steady contact.

### 4.2. Equivalent Shear-Strain Response Across the Friction Sweep

**Figure 5:**
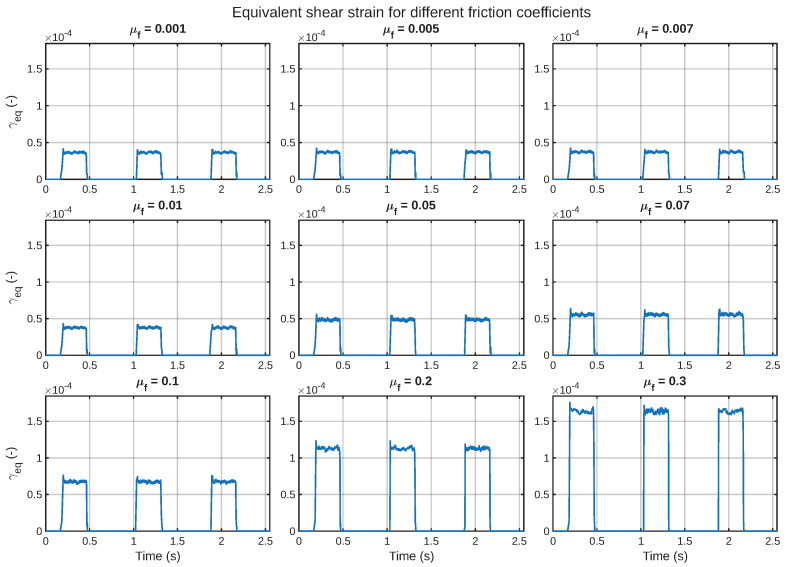
Equivalent shear-strain $\gamma_{\mathrm{eq}}$ with time for all friction values $\mu_{f}$.

The first mechanical result of interest concerns the equivalent shear strain, denoted by the symbol $\gamma_{\mathrm{eq}}$. This quantity acts as the mechanically imported field in the electrochemical analysis. Figure 5 displays the time histories of the full set of friction coefficients in the form of the equivalent shear strain, where the results are arranged in separate subplots with a common *y*-axis scale.

Some significant characteristics can be noted in the figure. Firstly, the shear response is localized in terms of time rather than being sustained over the entire period of the cycle. In other words, $\gamma_{\mathrm{eq}}$ is almost zero during the detached phases and only increases when the slider is in contact with the artificial skin. Secondly, the overall amplitude of the shear response is higher for higher friction coefficients. This suggests that the higher the friction between the two surfaces in contact, the higher the tangential stress transfer into the layered structure of the IPMC. Thirdly, the peak shear response is localized in the touch and sliding phases of the cycle, further reinforcing the dynamic nature of the mechanical excitation experienced by the sensor.

**Figure 6:**
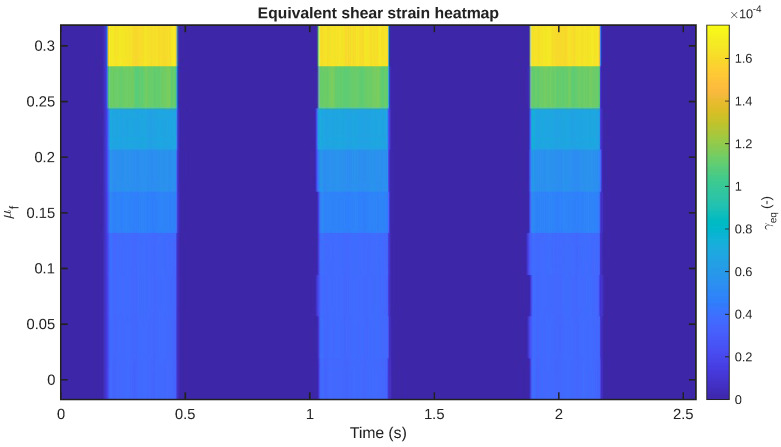
Heatmap representations of the shear-strain $\gamma_{\mathrm{eq}}$ response as a function of time and friction values $\mu_{f}$. Higher values are confined primarily to the contact-active portions of the cycle.

The same trend can also be observed in Figure 6, where the complete set of all $\gamma_{\mathrm{eq}}$ responses is presented as a time–friction heatmap. The figure offers a compact representation of the evolution of the shear field both with time and with the friction range. The concentration of the highest values in narrow time ranges again indicates that the mechanical stimulus is not distributed evenly over the whole range but, rather, depends on the sequence.

**Figure 7:**
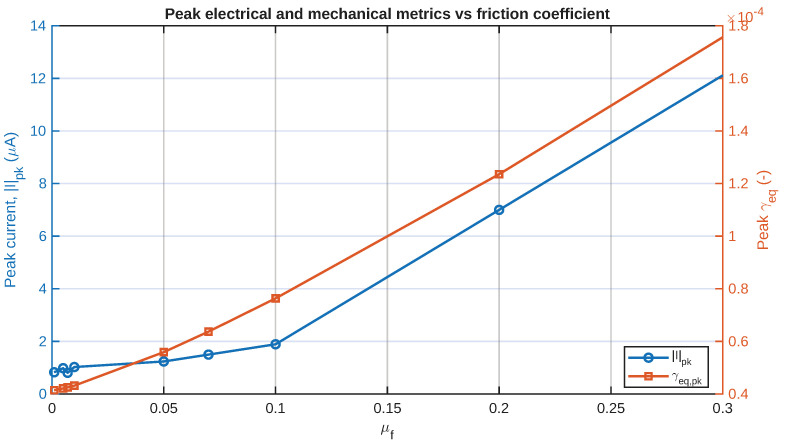
Peak electrical (short circuit current) and mechanical response metrics $\gamma_{\mathrm{eq}}$ as functions of the friction coefficient ($\mu_{f}$). The figure compares the peak current magnitude and peak equivalent shear strain across the full friction coefficients.

**Figure 8:**
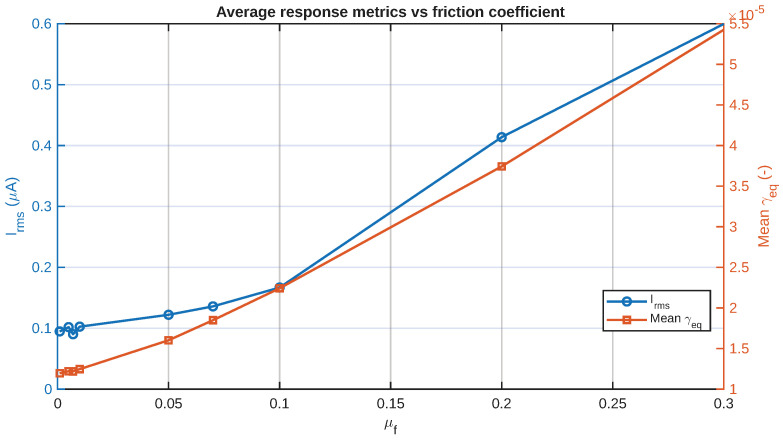
Average response as functions of the friction coefficient, consisting of RMS current and mean equivalent shear strain.

A more quantitative summary is given in Figure 7, where the peak equivalent shear strain is shown as a function of the peak current together with the peak current as a function of $\mu_{f}$. In addition, Figure 8 presents the average equivalent shear strain as a function of the RMS current. These figures are helpful in summarizing the time histories in a design-oriented way, making it easier to evaluate the monotonicity and relative sensitivity to friction.

Collectively, Figure 5, Figure 6, Figure 7 and Figure 8 illustrate that the friction coefficient does not merely scale a static deformation state. Rather, it alters the magnitude and duration of the applied shear stimulus that is conducted into the layered IPMC artificial skin. This is a critical consideration in understanding this sensor, because it is stimulated by an imported mechanically generated field.

Design-wise, the friction sweep shows two separate regimes of response behavior. In the low-friction regime, the tangential stress transmission through the IPMC layer is rather weak, leading to low $\gamma_{eq}$ and current generation levels. With the increase in the friction coefficient to the high-friction regime, the shear-stress transmission is mechanically much stronger, and the electrical response also rises faster. This suggests that the role of friction is not only to change the response level linearly but also to control the effectiveness of the conversion of the mechanical interaction into an electrical signal.

### 4.3. Predicted Current Response and Dynamic Sensing Behavior

**Figure 9:**
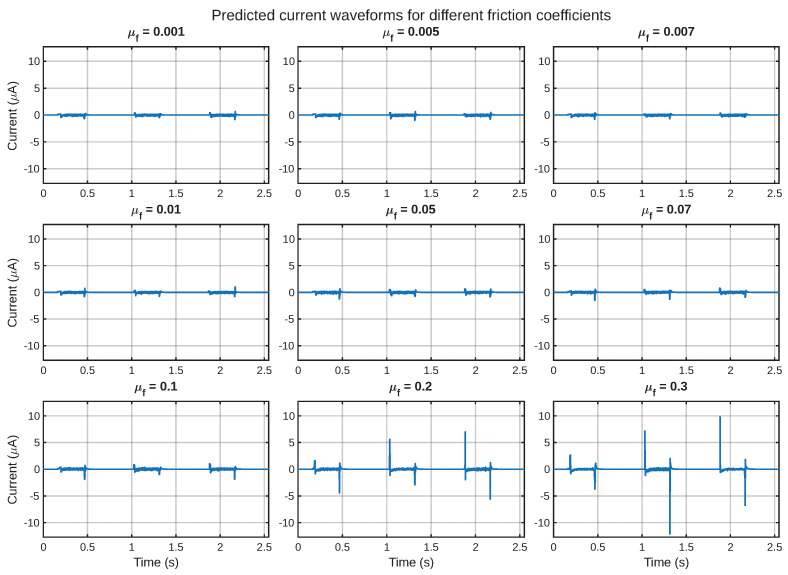
Predicted short-circuit current waveforms for all friction values.

The most significant result of this study, in terms of relevance to a potential application, is the transient current that is developed at the electrode boundary. Figure 9 displays a collection of predicted current waveforms for all friction coefficients, where a common vertical axis scale has been used. This type of common scale subplot is very useful, especially when it is required that a comparison of signal strength, waveform, and localization be made for all friction coefficients.

The nature of the current signals is also transient. In other words, instead of being large over the course of the motion cycle, they are concentrated over specific time intervals related to contact establishment, sliding activation, and separation. This also lends support to the view that this layered IPMC structure functions primarily as a dynamic tactile transducer, where currents are generated primarily due to time-varying mechanical excitations and reduce when the phases of detachment or weaker stimulation occur.

**Figure 10:**
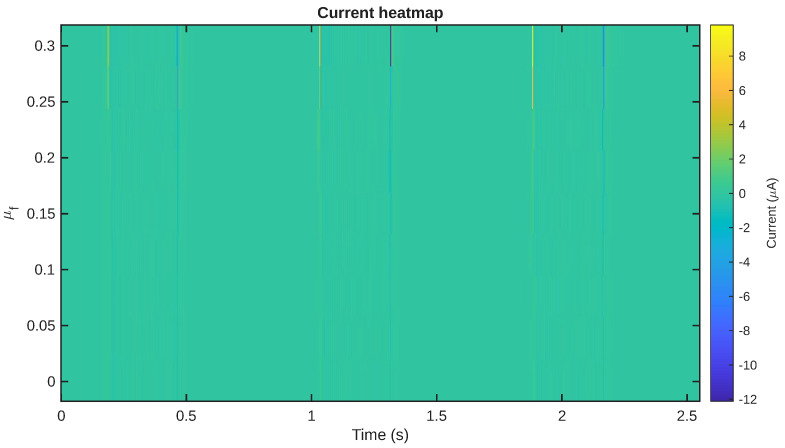
The representation of the predicted short-circuit current as a function of time and friction values. The largest signals are concentrated in the dynamically active contact stages of the cycle.

The nature of this assertion is further illustrated in Figure 10, which shows a time–friction heatmap of the currents. This figure shows that the highest currents are localized to the mechanically active regions of the signal, and that this is a friction-dependent effect. This is consistent with the mechanical results that showed friction-dependent enhancement of the imported shear field.

**Figure 11:**
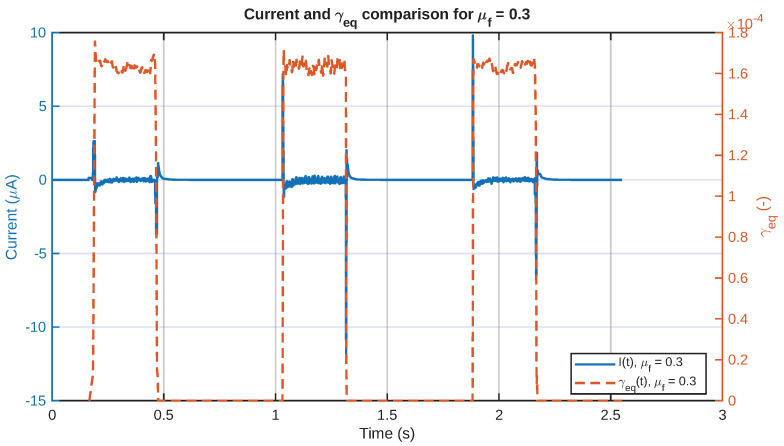
Comparison between the predicted current signal and the shear-strain waveform for a friction value = 0.3.

**Figure 12:**
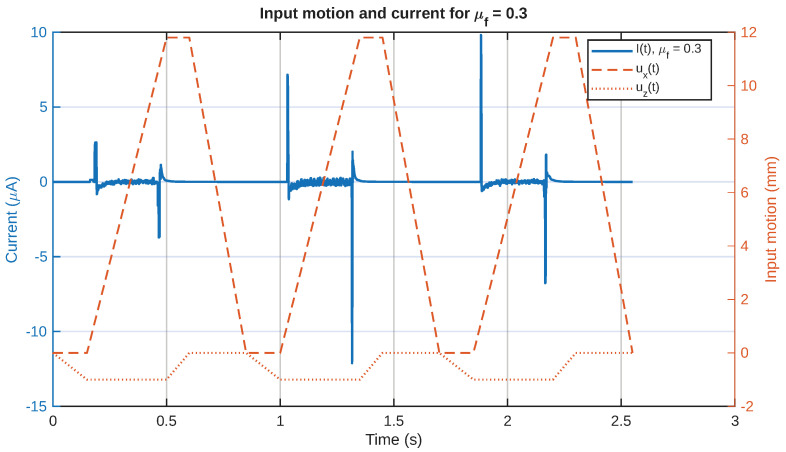
Comparison between the prescribed motion of the slider and the current signal for a friction coefficient = 0.3.

As a representative example of the relationship between the mechanical and electrical behavior, in Figure 11, the current signal and the equivalent shear-strain rate are plotted together for a chosen value of the friction coefficient. The relative position of the two signals in the figure demonstrates that the electrical signal has a strong relationship with the onset of the equivalent shear-strain rate field. Similarly, in Figure 12, the same representative current signal has been plotted together with the input motion, in order to demonstrate the relationship between the electrical signal and the touchdown and sliding stages, rather than the detached return stage.

**Figure 13:**
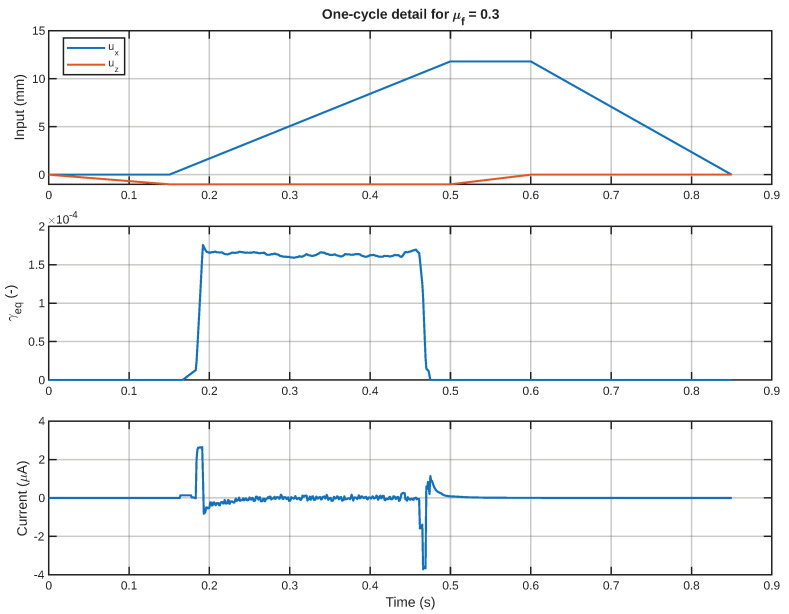
One -cycle motion view showing the imposed motion in the top plot, equivalent shear strain in the middle plot, and current signal for a friction value = 0.3 in the bottom plot.

**Figure 14:**
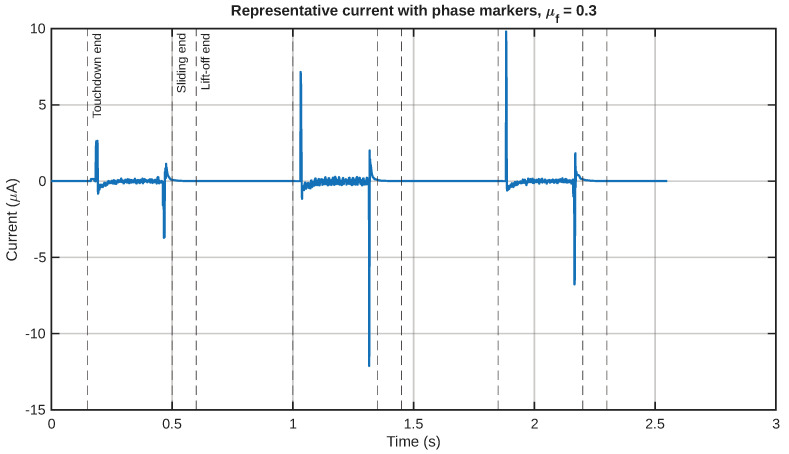
The current signal waveform annotated with cycle-phase markers indicating the end of touchdown, followed by the sliding and lift-off stages.

In order to better understand this response within one single cycle in more detail, Figure 13 shows the input motion, equivalent shear strain, and current signal over one single cycle. This figure is particularly helpful because it shows the relationship among input motion, mechanical stimulus, and output signal. Moreover, Figure 14 shows the boundaries of the phases of this single cycle directly on the corresponding representative current signal. This is helpful because this figure can assist in identifying which parts of this signal relate to touchdown, sliding, and lift-off.

**Figure 15:**
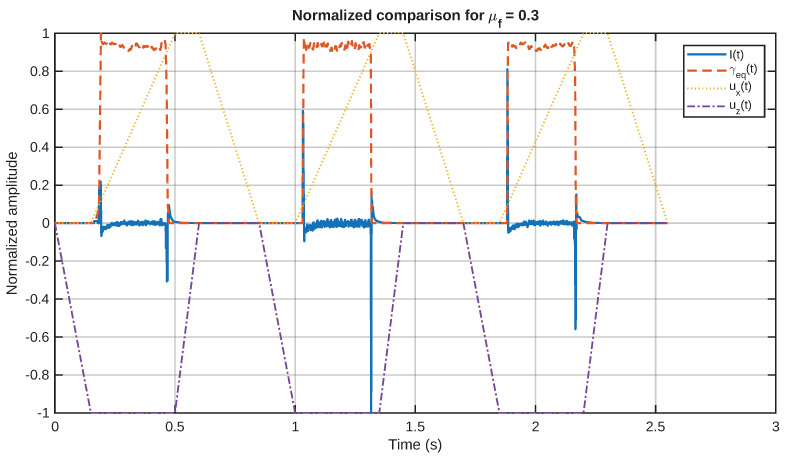
Normalized comparison among current, equivalent shear strain, and prescribed motion for a representative friction coefficient.

The normalized comparison of input motion, equivalent shear strain, and response is given in Figure 15. This plot is helpful because it reduces the effect of scale. In this normalized plot, it can be seen that the peaks in the current occur during the active phases of motion and reduce during the detached return period. This is consistent with the interpretation that the sensor response is dominated by transient mechanical events.

To summarize the electrical response more compactly, the peak current magnitude and RMS current are shown in Figure 7 and Figure 8. The results obtained here illustrate how friction can influence not only the characteristics of the signal’s shape but also the overall level of the resulting electrical signal. In practical terms, this means that friction can serve as a tuning parameter for signal detectability.

From the above observations, it becomes clear that the structure of the IPMC described above is best characterized as a dynamically operating shear-based tactile sensor. Specifically, significant peaks of activity are seen during stages where mechanical motion is involved, including initial contact, sliding while in contact, and detachment. In contrast, there is relatively little activity during the return stage, when the probe is not making physical contact.

### 4.4. Mechanical–Electrical Correlation

**Figure 16:**
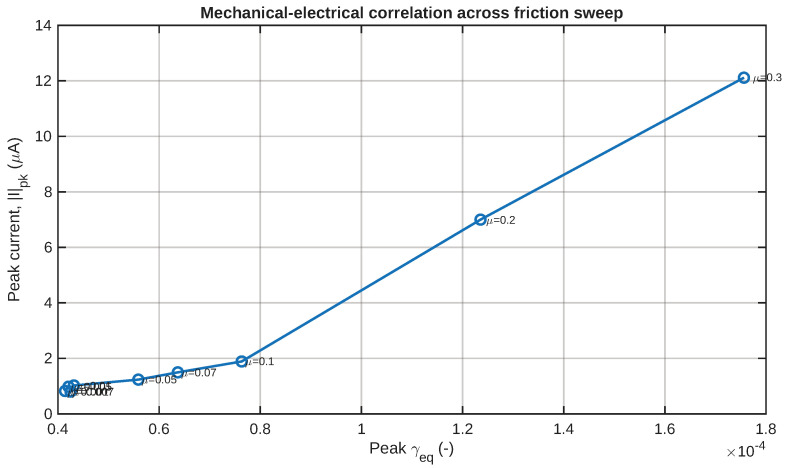
Correlation between peak current signal magnitude and peak equivalent shear strain across the friction values.

One of the key issues investigated through the proposed framework is the relationship between the electrical response and the shear field generated due to mechanical loading. This issue is effectively resolved in Figure 16, which illustrates the correlation between the peak electrical current amplitude and the peak equivalent shear strain for different friction values. In addition to the monotonic behavior and sensitivity discussed earlier based on Figure 7 and Figure 8, this figure allows one to assess the practical implications of the proposed framework.

As can be seen from the rise pattern in Figure 16, there seems to be a positive relationship between increased mechanical shear transmission force and higher electrical output transients, supporting the shear-to-electrochemical interaction principle chosen for this work. This implies that the contact condition determined by the coefficient of friction regulates both the magnitude of applied shear and the possibility of capturing an electric signal generated thereby. Despite its theoretical nature, this relationship may be considered helpful for the purposes of system optimization in design.

The importance of the correlation plot is that it offers a compact link between contact mechanics and tactile sensing performance. A smooth and rising trend is consistent with the idea that higher friction-enhanced shear transfer strength corresponds to higher strength in current-based sensing output. Although not necessarily linear, a systematic trend is all that is required to support the use of this artificial skin made of layered IPMCs as a designable shear-sensitive tactile transducer.

### 4.5. Implications for Robotic Tactile Interfaces

From an application viewpoint, the results obtained here suggest that interfacial friction is not only a boundary condition but also an effective parameter for tuning tactile sensing in layered IPMC structures. In robotic devices, friction can depend on various factors, such as surface textures, applied normal loads, local compliance, contamination, and environmental factors. In this work, it is demonstrated that the model can account for such variations not only in terms of mechanical shear field but also in terms of transient currents. This also indicates that layered IPMC structures can not only sense contact but also possibly detect friction-dependent aspects of tactile interactions.

On the other hand, the results also show that the most informative regime is not necessarily the regime with the largest friction coefficient. Although the overall tangential loading can be increased with higher friction coefficients, the interfacial constraint may also result in more distinct waveform features and nonlinearity. A practically useful regime window is thus likely to be needed for robotic tactile interpretation in terms of signal amplitude, waveform clarity, and robustness of interpretation.

In summary, the present findings support the idea that this multi-layered artificial skin made of IPMC can be used as a current-based tactile transducer for cyclic touch–slide–lift interactions. In this sense, this type of analysis that is carried out at a mechanical and electrochemical level can be a physically grounded approach to explore how interaction at the surface of a robot can be translated into an electrical signal.

### 4.6. Model Validity, Limitations, and a Strategy for Future Validation

This work relies exclusively on simulation and must thus be viewed as an exploratory framework for developing a physics-based model, and not as a quantitative model whose validity has been verified to any extent. The electrochemical model employed, along with current measurement and transient response considerations, is in agreement with the previous literature on IPMC sensory modeling approaches. The new application-oriented scenario presented in this work (touch–slide–lift) represents a case that has yet to receive experimental validation.

Some key limitations need to be mentioned in this regard. Firstly, the relationship between the shear strain and fixed-charge density is established here as a phenomenological coupling equation and has yet to be experimentally calibrated. Secondly, the frictional contact interaction has been described via the Coulomb contact interaction model, which neglects the influence of surface roughness, adhesion, varying hydration levels, and material degradation. Finally, the parameters were taken as baseline values for simulation purposes only, with no consideration of their inherent variability.

Nevertheless, the model provides a physically based pathway from sliding friction contact to transient current generation and exhibits behaviors that are physically plausible for a time-varying process such as IPMC sensing described in the previous literature. A logical next step would be to conduct a controlled experiment in which the layered IPMC structure undergoes reciprocating sliding contact while the current output is measured. This would give insight into whether the generated current is timed and polarized as expected, as well as providing the necessary feedback to calibrate the coupling constant in the model.

One more drawback of the current approach is the use of the Coulomb type of friction law as an interface contact approach. Even though such an approach can be applied appropriately as a first-order approximation for sensitivity analysis based on the friction, no consideration has been made regarding any rate-dependent properties, adhesion effects, roughness effects, or other non-ideal phenomena occurring at the interface level. Therefore, any more complex friction models could constitute an excellent extension to the current study.

## 5. Conclusions

In this work, a 3D finite element and PNP model of a layered IPMC structure in the context of cyclic touch, slide, and lift of a finger-like object over the IPMC-based artificial skin has been presented. By incorporating the frictional contact mechanics with the electrochemical transport in the Nafion core of the IPMC structure, the model was able to predict the current generated in the structure. It has been shown that the electrical response is localized only in the mechanically active stages of the object’s interaction with the IPMC structure.

The friction sweep results show that the higher the friction between the interfaces, the larger the equivalent shear-strain response and the current output. The mechanical–electrical correlation observed in the simulation results confirms the feasibility of using the IPMC-based multi-layer structures as friction-sensitive artificial skins in robotic tactile devices. Although this work is only numerical, it provides a physically meaningful basis for the analysis and design of IPMC-based tactile skins. Future studies will aim at validating the results, improving the coupling formulation, and further optimizing the sensor configuration for robotic tactile devices.

In the current phase of development, the model can be viewed as a phenomenological coupling of simulations in shear-sensitive tactile perception during sliding contact, with possible applications in identifying initial slip, and with experimentally verified results to follow in subsequent phases.

## Figures and Tables

**Table 1:** Baseline geometry, loading conditions, and model parameters used in the COMSOL simulations for the layered IPMC artificial skin under cyclic touch–slide–lift motion.

Category	Parameter	Value (Units)
**Geometry (IPMC)**	Length *L*	15 mm
	Width *W*	2 mm
	Bottom electrode thickness $t_{b}$	0.005 mm
	Nafion core thickness $t_{n}$	0.190 mm
	Top electrode thickness $t_{t}$	0.005 mm
	Total IPMC thickness $t_{\mathrm{IPMC}}$	0.200 mm
**Geometry (slider)**	Slider length $L_{s}$	3 mm
	Slider width $W_{s}$	1.6 mm
	Slider height $H_{s}$	0.5 mm
	Initial gap $g_{0}$	1.0 mm
	Edge offset *e*	0.1 mm
	Sliding stroke *s*	11.8 mm
**Cyclic motion**	Touchdown time $t_{D}$	0.15 s
	Sliding time $t_{S}$	0.35 s
	Lift-off time $t_{L}$	0.10 s
	Detached return time $t_{R}$	0.25 s
	Total cycle duration $T_{\mathrm{cyc}}$	0.85 s
	Number of cycles $n_{\mathrm{cyc}}$	3
	Total simulation time $t_{\mathrm{end}}$	2.55 s
	Additional preload $\delta_{p}$	0.002 mm
**Mechanics (Nafion/IPMC core)**	Density $\rho_{s}$	2130 kg/m^3^ [35]
	Young’s modulus *E*	$2.0\times 10^{8}$ Pa
	Poisson ratio ν	0.49
**Contact**	Contact law	Coulomb friction
	Friction coefficient $\mu_{f}$	sweep: 0.001–0.30
	Contact formulation	Penalty
	Manual tuning factor	0.05
**Electrochemistry (PNP)**	Temperature *T*	290 K [35]
	Valence *z*	1
	Diffusion coefficient *D*	$3.2\times 10^{-11}$ m^2^/s [35]
	Mobility $u_{m}$	$1.28\times 10^{-9}$ m^2^/(V s)
	Permittivity ϵ	$5\times 10^{-3}$ F/m [35]
	Initial cation concentration $c_{0}$	1056 mol/m^3^ [35]
	Coupling coefficient α	0.001
**Time stepping**	Time range	range(0, 0.001, t_end_)

## Data Availability

The original contributions presented in this study are included in the article. Further inquiries can be directed to the corresponding author.
